# Tectonostratigraphy of the Jurassic accretionary prisms in the Sikhote-Alin region of Russian Far East

**DOI:** 10.1038/s41598-021-98748-5

**Published:** 2021-09-29

**Authors:** Vladimir V. Golozubov, Ludmila F. Simanenko

**Affiliations:** Far East Geological Institute of Far Eastern Branch, Russian Academy of Sciences, Prospect 100-letiya, 159, Vladivostok, Russia 690022

**Keywords:** Ocean sciences, Solid Earth sciences

## Abstract

We propose a scheme to subdivide the Samarka terrane, a Jurassic accretionary prism fragment, into tectonostratigraphic complexes. This subdivision provides a basis to study these formations and map them on a medium- to large-scale. Each complex corresponds to a certain stage in the accretionary prism formation. Thus, the complexes composed of subduction mélange and olistostromes (in our case, Ust-Zhuravlevka and Sebuchar complexes), can be correlated to episodes when the underthrusting of seamounts hampered subduction, as evidenced by seamount fragments contained in the complexes. Episodes of relatively quiet subduction have also been identified, resulting in complexes composed mainly of normally bedded terrigenous and biogenic formations (Tudovaka and Udeka and, partially, Ariadnoe complexes). Particularly considered is the Okrainka-Sergeevka allochthonous complex – a fragment of continental plate overhanging a subduction zone. It was included in the accretionary prism during gravitational sliding on the internal slope of the paleotrench. All volcanic rocks in the accretionary prism are allochthonous fragments of the accreted oceanic crust. The absence of the Jurassic-Berriasian volcanic belt related to this prism, as well as synchronous autochthonous volcanism, indicates that the Samarka terrane accretionary prism formed under conditions of flat-slab subduction, similar to modern examples along the Andean margin.

## Introduction

An accretionary prism (or wedge) is an important structural element in the convergent boundary between continental and oceanic plates. Contemporary accretionary prisms (Cascadia, Barbados, Nankai, etc.) are made up of imbricated sheets, inclined toward a continent or an island arc and become younger oceanward^1–4^. An accretionary prism is bounded by a basal detachment surface (décollement) located within the sedimentary section which sometimes penetrates into lower horizons and down to the ocean crust basalt. On seismograms, décollement surfaces can be clearly traced for tens of kilometers from away a deep sea trench^5–7^. Repeated imbricates are composed of abyssal plain, trench, continental slope and shelf deposits. The formation of these imbricate structures is assumed to be directly related to the downward movement of increasingly younger fragments of an oceanic plate under a continental plate or an island arc, which is an integral component of subduction^2,8–12^.

**Figure 1:**
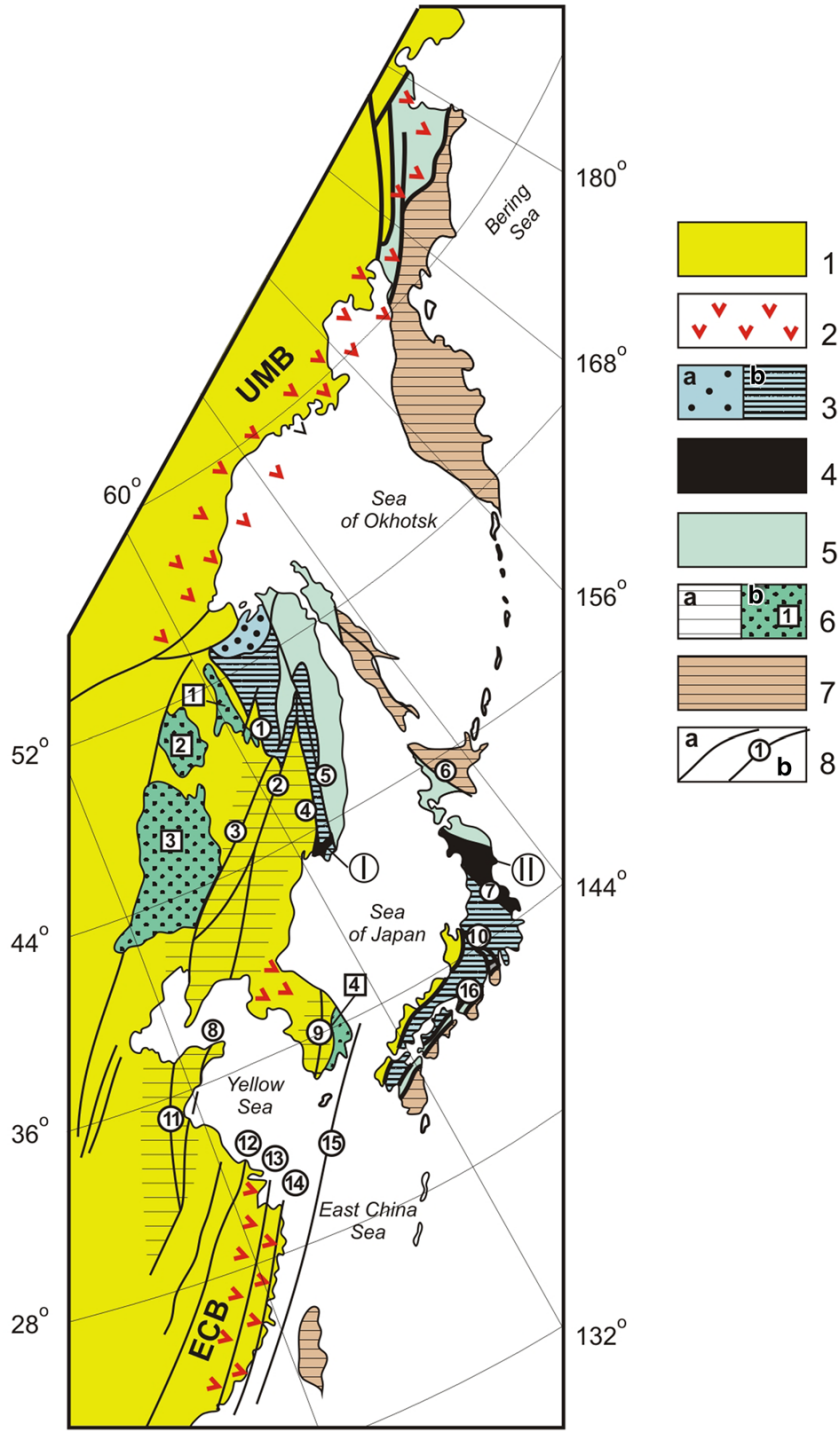
Distribution of the Jurassic and Early Cretaceous complexes along the north-western continental framing of the Pacific Ocean. Modified after [^14^, fig. 1]. 1 – Pre-Jurassic terranes; 2 – volcanic belts of Late Jurassic-Early Cretaceous active continental margin: UMB – Udsko-Murgalsky, ECB – Eastern China; 3 – Jurassic terranes – fragments of margin-continental turbidite basin (**a**) and accretionary prism (б); 4 – fragments of Pre-Cambrian-Early Paleozoic continent detached from the eastern margin of Asian continent and included in Jurassic accretionary prism structure: I – Okrainka-Sergeevka complex, II – Abakuma and South Kitakami terranes; 5 – Early Cretaceous terranes; 6 – areas of existence of Jurassic and Early Cretaceous epicontinental strike-slip basins (**a**), the largest of these basins (**b**): 1 – Bureya, 2 – Seya-Bureya, 3 – Songliao, 4 – Kensan; 7 – Late Cretaceous and Cenozoic terranes; 8 – faults and fault zones (**a**), including left-lateral strike-slip faults and fault zones of Tan-Lu Wrench Fault System (**b**): 1 – Kukansky, 2 – Dunhua-Mishan (Alchan), 3 – Ilan-Itun, 4 – Arsen’evsky, 5 – Central Sikhote-Alin, 6 – Hidaka, 7 – Tanakura, 8 – Yalu Jiang-Quindao, 9 – Kwangju-Yondong, 10 – Fossa-Magna, 11 – Tan-Lu, 12 – Tienmushan-Bajishan, 13 – Lishui-Haifong, 14 – Changle-Nanao, 15 – Korea-Taiwan, 16 – Median Tectonic Line.

Fragments of ancient accretionary prisms are recognized slightly later, primarily along the northern and northwestern portions of the Pacific coast, where they form an almost continuous chain of Mesozoic and Cenozoic orogenic belts^10–18^ (Fig. 1). Accretionary prisms may include slices of ophiolites – serpentinite mélange with rock clasts from different layers of ocean plate stratigraphy – harzburgite, gabbroid, basalt, limestone and chert^19–25^. It is assumed that these ophiolite lithologies are fragments of accreted mid-ocean ridges^19,21^ or the melanocratic basement of island arcs, seamounts and guyots^21,26^.

Fragments of accretionary prisms composed mainly of terrigenous and tuffaceous-terrigenous rocks, which accumulate along the continent or island arc before being drawn into a subduction zone, are most common. A typical example is the modern prism of Cascadia^1,4^. It can be assumed that these prisms formed if subduction occurred without hindrance.

It is a different matter when oceanic plateaus, chains of seamounts and guyots enter a subduction zone. A guyot is a giant, underwater volcanic mountain with a flat top more than ten kilometers in diameter. The Guyot may have a base diameter greater than 100 km and rise over 4 km above the seafloor. The emplacement of such a large structure in a subduction zone is obviously long and complex. It includes a jump of the subduction zone, gradual destruction of the volcanic edifice and its carbonate "cap" and mélanging of recently deposited terrigenous rocks. The process creates compressional structures and clay diaprism. Slowdown of subduction is likely accompanied by opposite-directed movement of the overhanging plate. Blocks moving from the continent or an island arc, as well as previously accreted “oceanic” fragments, gravitationally slide down the slope front, leaving a trail of their destruction products. This phenomenon creates synsedimentary tectonic nappes and associated olistostrome horizons in accretionary prisms^14,27,28^. In this case, the accretionary prism consists of varying degrees of tectonized, terrigenous, clayey-siltstone matrix containing (a) interlayers turned into lenses (boudins) and inclusions, lumps and slices of autochthonous, intra-basin sandstone and siltstone sediments, (b) allochthonous fragments of the overhanging plate and (c) allochthonous inclusions, blocks and sheets of accreted ocean plate strata fragments, primarily pelagic chert, seamount basalt and caps of limestone from the top of paleogayots^21–26^.

These structural features sharply distinguish fragments of accretionary prisms within sedimentary formations, and must be taken into account when studying and mapping^29^. This is because the “Stratigraphic Code”, Steno's law which states that deeper layers must be older than layers closer to the top of existing section^30^, is practically inapplicable in accretionary prisms. The “Code” assumes that rock dislocations take place after their deposition. As mentioned, parts of accretionary prisms show co-sedimentational tectogenesis, including gravitational tectogenesis, and disturbances in the age of sedimentary succession. Often, clearly expressed thrusts are not found at the base of overlying older formations.

In view of the above, different approaches to studying and mapping ancient accretionary prisms fragments have emerged. Thus, when studying the Samarka terrane (South Sikhote-Alin), it was proposed to divide all its stratigraphic units not into formations and series, but into tectonostratigraphic complexes, each corresponding to an episode of the subduction process^14,15^. These principles have thus far not attracted enough attention nor found practical application, since they were developed after the Far East territories were mapped geologically at a scale of 1:200,000 and, as in the case of the Sikhote-Alin and Japan Islands areas, 1:50 000. The mapping was conducted in strict accordance with the Stratigraphic Code^30^, that is, without considering the role of tectonism in the formation of complexes. Hence, the geological maps provide a distorted view of the geological structure and history of the territories. Any reconsideration and improvement of the compiled maps is hindered by a lack of financial resources and highly skilled specialists. Nevertheless, we decided to summarize the available data on the structure of the well-studied Samarka terrane (South Sikhote-Alin), a fragment of the Jurassic accretionary prism, and propose a possible mechanism of prism formation. We also hope that the principles we have developed for the subdivision of accretionary prism fragments will be useful in further studies of transition zones between continents to oceans, both in our country and abroad.

## Tectonostratigraphic complexes of the Samarka terrane, Southern Sikhote-Alin

**Figure 2:**
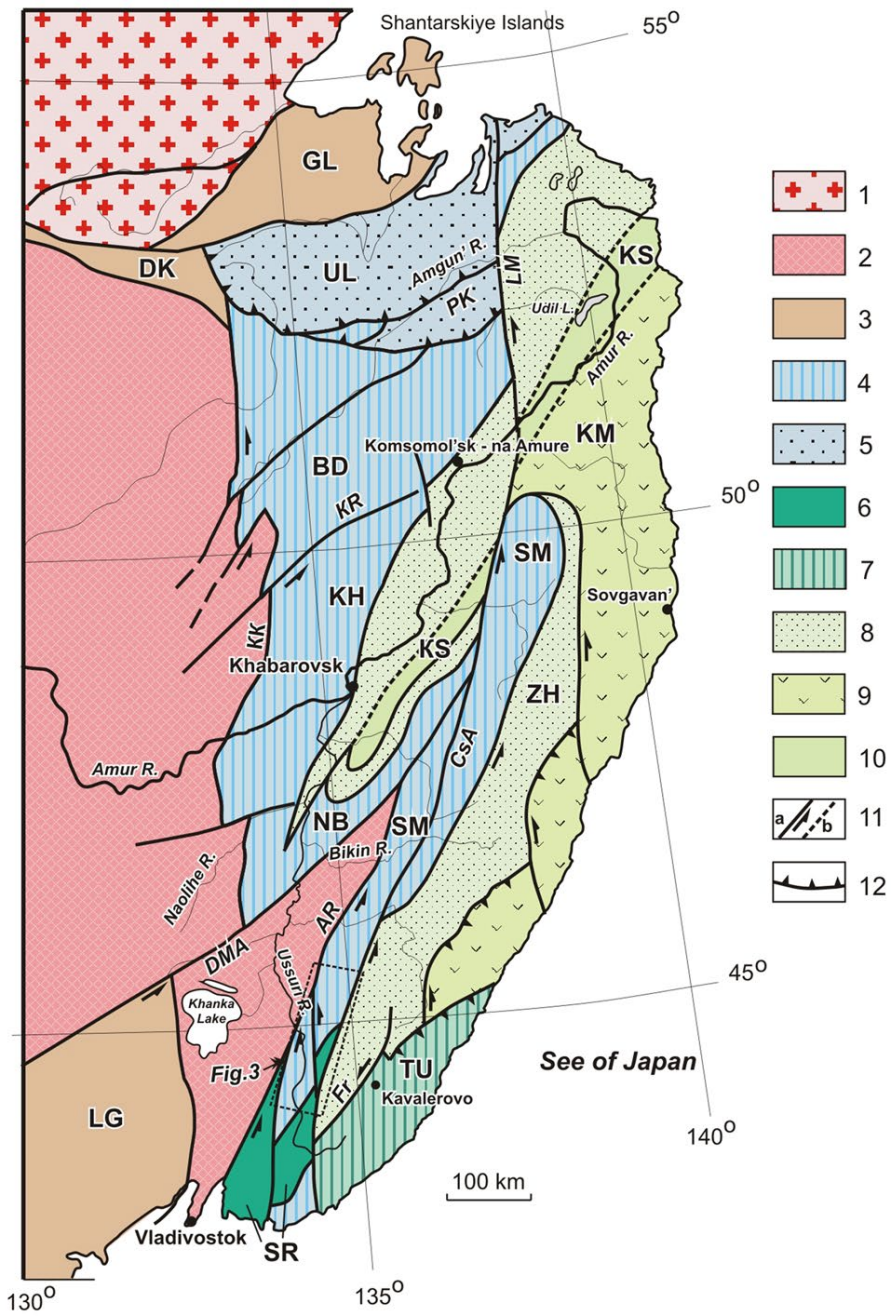
Terranes of the Sikhote-Alin orogenic belt and adjacent territories. Modified after [^14^, fig. 7]. 1–2 – Pre-Cambrian-Early Paleozoic structures: 1 – North-Asian Craton, 2 – Bureya-Jiamusi-Khanka Superterrane; 3 – Paleozoic terranes: – Dzhagdy-Kerbi (DK), – Galam (GL),– Laoelin-Grodekovo (LG); 4–5 – Jurassic terranes: 3 – fragments of accretionary prisms (SM – Samarka, NB – Nadanhada-Bikin, KH – Khabarovsk, BD – Badzhal), 5 – fragments of near-continental turbidite basin (UL – Ulban); 6 – fragments of the Pre-Cambrian-Early Paleozoic continent, involved in Jurassic accretion wedge structure and subjected together with them to cycle sin- and postaccretional deformations (SR – Okrainka-Sergeevka complex of the Samarka terrane); 7–10 – Early Cretaceous terranes – fragments of: 7 – Neocomian accretionary prism (TU – Taukha terrane), 8 – near-continental turbidite strike-slip basin (ZH – Zhuravlevka terrane), 9 – Barremian-Albian island arc system (KM – Kema terrane), 10 – Albian accretionary prism (KS – Kiselevka-Manoma); 11 – left-lateral strike-slip faults established (**a**) and supposed (**b**) including: КК – Kukansky, KR – Kursky, LM – Limurchansky, DMA – Dunhua-Mischan (Alchan), AR – Arsenievsky, CSA – Central Sikhote-Alin, FR – Furmanovsky Faults; 12 – thrusts (PK – Paukansky).

The Samarka terrane, a fragment of the Jurassic accretionary prism^13,15,31–33^, forms a 30–70 km-wide strip extending north-northeastward for nearly 900 km from the southern coast of Primorye to the Amur River basin (Fig. 2). The terrane is bounded in the east by the Central Sikhote-Alin Fault and one of its branches and in the west by the Arsen’ev Fault. North of the Bikin River valley, a continuation of the Samarka terrane is called the Nadanhada^17^ or Nadanhada-Bikin^15^ terrane. On the left bank of the Amur River’s lower course, continuations of the Samarka terrane are named the Khabarovsk and Badzhal terranes^34^ (Figs. 1, 2). The Tamba-Mino-Ashio and other terranes, which form the large portion of the Japan islands^16,18,25^ are also considered continuations of the Samarka terrane^14,17,35^ (Figs. 1, 2). Accretionary prism fragments of similar composition and age are traced further south along the Pacific margin to the island of Borneo^17^.

The Samarka terrane mainly consists of arkosic siltstone and silty mudstone terrigenous rocks. These matrix-forming rocks contain blocks (including exotic rocks) of varying composition, age and genesis. Such formations, usually described as “sedimentary mélanges, olistostromes, or horizons of landslide breccia”, are typical of suprasubduction accretionary prisms and are described in detail in numerous publications (e.g.,^16,25,36^). In addition, we traditionally distinguish a special class of co-sedimentary tectonites (hydrotectonites), – rocks deformed during sedimentation or initial diagenesis. They are represented by siltstones and silty mudstones with interlayers of sandstone and chert. The latter are deformed to some extent and often transformed into boudins with dispersed sandy material in their extensions. Unlike common tectonites, the clayey material of hydrotectonites containing boudins have a massive structure with no slip traces, filling transverse fractures in boudins. Transitions from sandstone boudins to the host clayey rock are often gradual. Such structures can form when weakly lithified sandstone interlayers are deformed, and the host clayey rock is in a semiliquid state^27,28,37,38^. Identifying these structures is not always easy, since subsequent dislocations cause the features listed above to disappear and the rocks appear as common tectonites. This forms monotonous tectonite sequences many hundreds of meters thick. A possible genetic relationship between hydrotectonites, closely associated olistostromes and the gravitational sliding of allochthonous plates is discussed below in the "Discussion" section.

The subdivision of the Samarka terrane into tectonostratigraphic units is demonstrated in a series of publications by I.V. Kemkin and coauthors^15,31,39^. Specifically, in the middle basin of the Ussuri River, I.V. Kemkin^15^ identified two subterranes, the lower – Eldovaka and the upper Sebuchar by dating radiolarian and conodont remnants. In the Eldovaka subterrane, the sedimentary rock matrix age is Middle-Late Jurassic, and the matrix of the overlying Sebuchar subterrane is Lower-Middle Jurassic. When subdividing the accretionary prism, the abovementioned authors concentrated on the age of the chert and terrigenous transitional layers, since this transition is believed to reflect when pelagic oceanic sediments approached the subduction zone. Accordingly, this records the age of the plunging plate and time of subduction. It is important to note that the transition layers form extremely rare allochthonous sheets in accretionary prisms; usually, such sheets are composed of a single rock type, primarily chert. To date, five sections have been studied in detail in Sikhote-Alin and a sliding time is observed between the end of chert accumulation in the Lower Jurassic Toarcian and the Upper Jurassic Oxfordian stages^39^. The integration of these five widely dispersed sections, which stretch from the northern Katen River basin to the upper reaches of the southern Ussuri River into a single tectonostratigraphic succession characterizing the accretionary prism structure (as it is done by I. Kemkin and A. Filippov^39^), in our opinion, cannot be considered correct. The sections were united purely mechanically, without any structural evidence. The tectonostratigraphic column proposed by these authors was compiled by postulating that the prism formed by the downward movement of progressively younger oceanic plate fragments. Therefore, the given tectonostratigraphic succession cannot be a confirmation of the chosen model. Moreover, with this approach, the material characteristics of complexes important for geological mapping, including the presence or absence of synsedimentary tectonites at different levels of the prism are relegated to the background. The composition, age, geodynamic nature of allochthonous material and their role in geological succession are not considered.

We prefer the scheme we created to subdivide the Samarka terrane into tectonostratigraphic complexes, which takes into account data on the composition, age, structure and quantitative proportions of both the matrix rocks and allochthon material^13,14,32^. This designates each complex as a tectonic slice lying at a certain structural level, which differs from underlying and overlapping complexes in rock composition, degree of tectonic reworking and age. These differences allow us to confidently distinguish boundaries between complexes. Each complex is given its own name, and its age is determined by dating the terrigenous matrix. Large *exotic* blocks and slices of more ancient rocks included in the matrix can be shown on maps and charts, both in-scale and out-of-scale, using individual signs or colours in accordance with their age.

The upper course of the Ussuri River and its right reaches, the Zhuravlevka, Pavlovka and Malinovka rivers were chosen as the reference site for our terrane division schemes because detailed studies of the local complexe structure leave no doubt about their structural position. In addition, there are sufficient age dates of both the matrix and allochthonous bodies.

## Tectonostratigraphic complexes in the upper Ussury River basin

**Figure 3:**
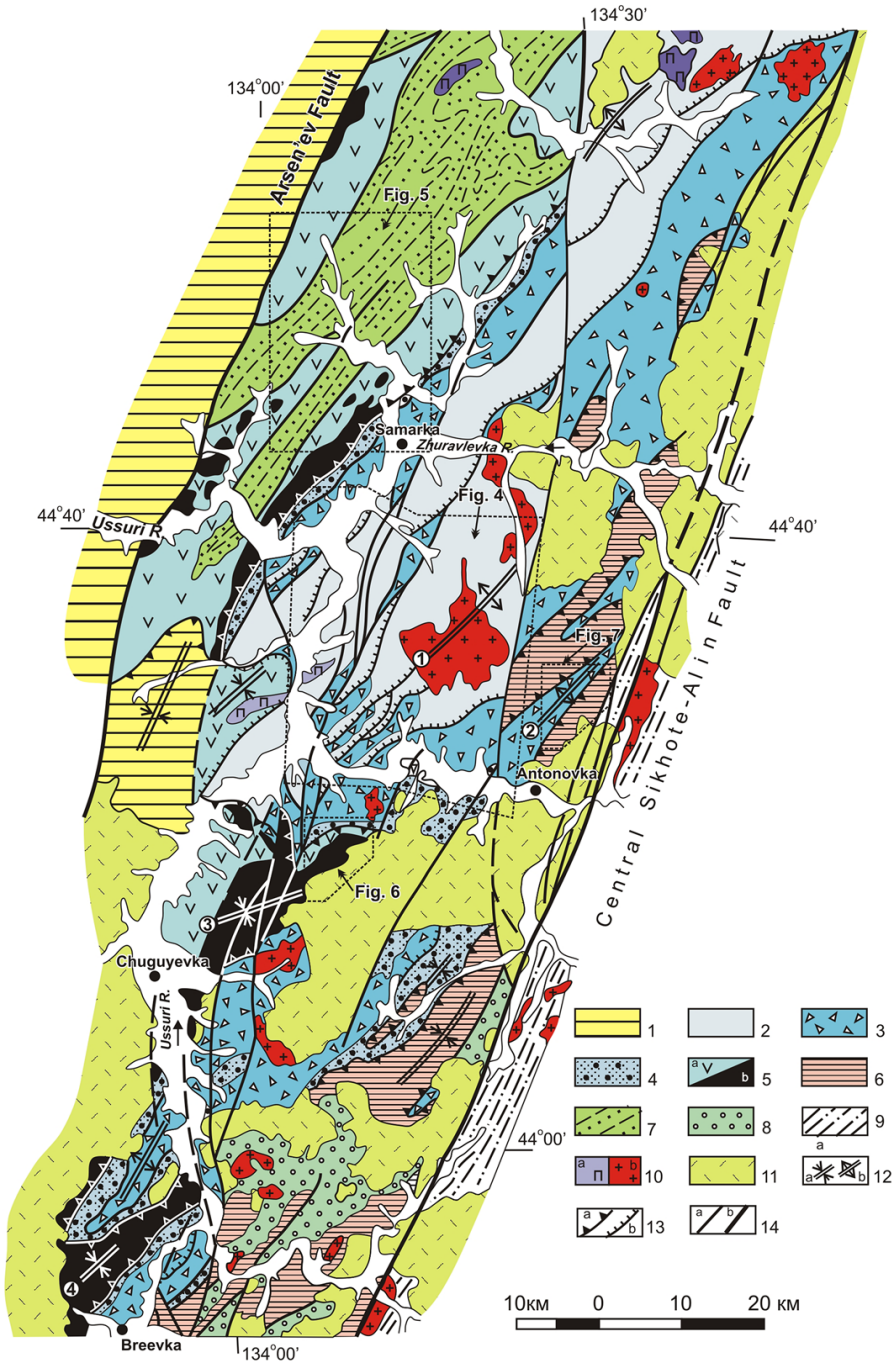
Geological setting of south part of Samarka terrane in the upper part of Ussuri River basin. Modified after [^27^, Fig. 3]. Location see in the Fig. 2. 1 – Upper Permian and Mesozoic cover complexes of the Khanka superterrane; 2–7 – Jurassic complexes of the Samarka terrane : 2 – Tudovaka complex (siltstone, chert, basalt), 3 – Ust'-Zhuravlevka complex (mixtite with the siltstone matrix, sandstone–siltstone alternation, allochthonic plates and blocks of chert, basalt and limestone), 4 – Udeka complex (sandstone–siltstone alternation), 5 – Sebuchar complex (a – mixtite with basalt blocks, b – ultramafic-gabbro unit of the Kalinovka ophiolytic complex, 6 – Okrainka-Sergeevka allochthonic complex (intrusive and metamorphic rocks of the Pre-Devonian basement, overlapped by the Permian, Triassic and Jurassic cover complex), 7 – Ariadnoe complex (sandstone–siltstone alternation, mixtite); 8 – Early Cretaceous cover (shallow-marine terrigenous deposits); 9 – Zhuravlevka terrane (Lower Cretaceous turbidite); 10 – Lower Cretaceous gabbro and pyroxenite (**a**) and granite (**b**); 11 – Upper Cretaceous volcanics; 12 – fold axes: sinform (**a**), antiform and anticline (**b**) including: Koksharovka-Malinovka antiform (1), Vasil'ev antiform (2), Chuguyevka (3) and Breevka (4) sinforms; 13 – thrusts: a – Jurassic sinsedimentational, b – Early Cretaceous sinfolded; 14 – left-lateral strike-slip fault (**a**), including boundaries of terranes (**b**).

According to available data, the Jurassic accretionary prism in the study area can be differentiated into six, sheet-shaped tectonostratigraphic complexes. From bottom to top these are: Tudovaka, Ust-Zhuravlevka, Udeka, Sebuchar, Okrainka-Sergeevka and Ariadnoe (Fig. 3). In the Early Cretaceous, the above complexes were deformed by NNW-directed regional compression after subduction was replaced by left-lateral sliding of the Isanagi oceanic plate along the continental edge^13,14,32^***. ***This NNW-directed compression reactivated this imbricate series of under-thrusted tectonic sheets that were formed during subduction, further complicating the overall structure. For this reason, Early Cretaceous folding in this area has been reduced, and some sections with low-angle dips of 10–20° are preserved in the core of the largest Koksharovka-Malinovka antiform. The ensemble of Cretacous structures is superimposed by left-lateral, strike-slip faults of NNE and meridional trends with sinistral displacements up to 40 km^40^.

### The Tudovaka complex

**Figure 4:**
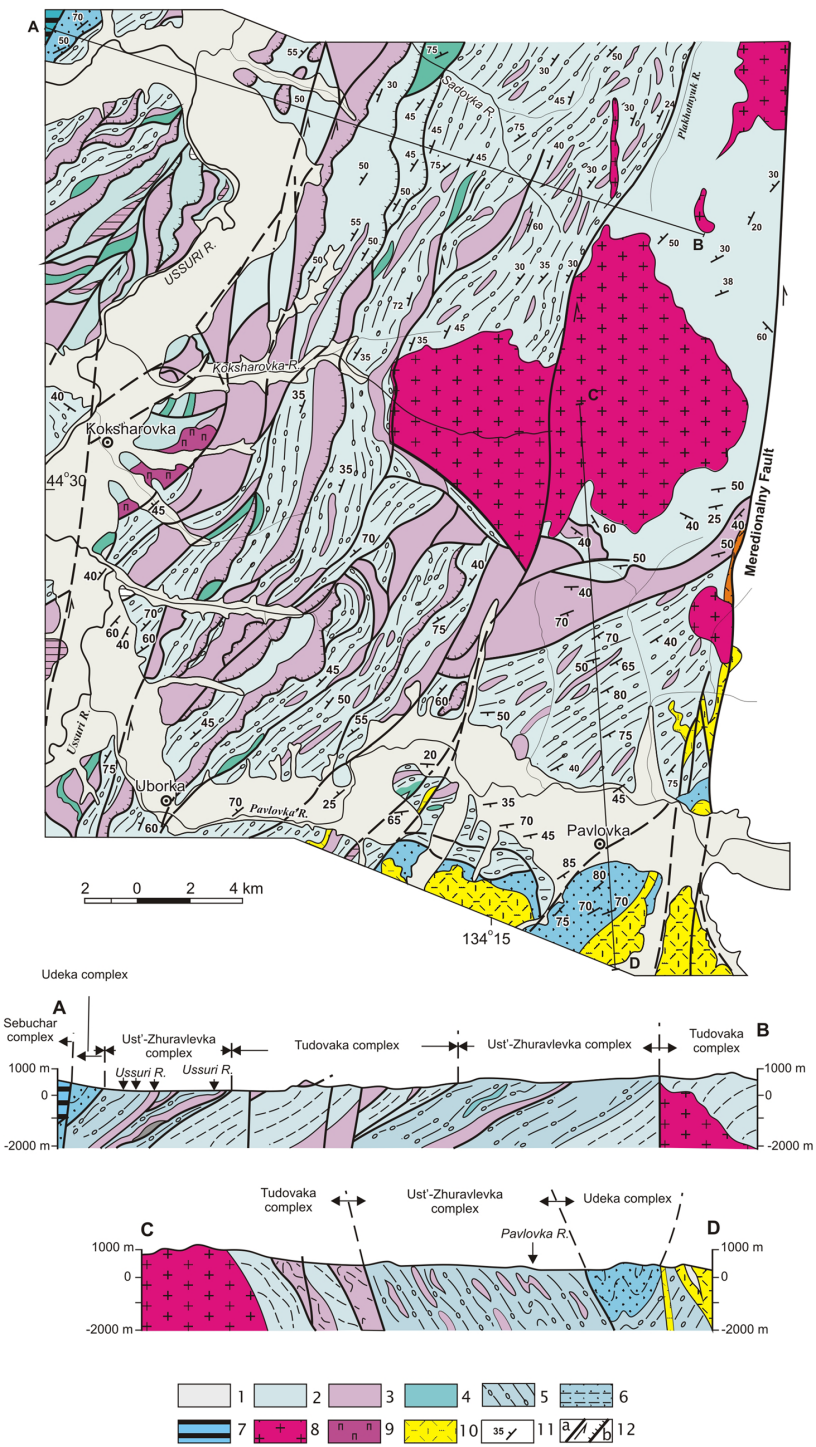
Lower structural levels of the Samarka terrane on the right bank of the Ussuri River and near mouth of the Pavlovka River. Modified after [^14^, Fig. 9]. Location see in the Fig. 3. 1 – Quaternary alluvium; 2 – siltstone with the rare sandstone interbeds; 3 – chert and siliceous argillite; 4 – basalt; 5 – mixtite with siltstone matrix, included the sandstone interbeds, and also blocks and slices of sandstone and chert, more rarely basalt and limestone; 6 – sandstone–siltstone alternation; 7 – gabbroid of the Kalinovka complex; 8 – Early Cretaceous granite; 9 – Early Cretaceous piroxenite; 10 – Late Cretaceous volcanics; 11 – bedding; 12 – faults: a – strike-slip fault, b – thrust.

Occupies the lowest structural position in the axial part of the Koksharovka-Malinovka antiform (Figs. 3, 4). A package up to 800 m thick of flaggy siltstone, silty mudstone and rare interbeds of sandstone is mapped in the core of this structure. This is overlain by sheets of similarly flaggy siltstone alternating with complexly dislocated siliceous rocks, sometimes in association with basaltic volcanic rocks. The basalts are characterized by high Ti (TiO_2_ > 2,5%), Mg (MgO > 7%) and K (K_2_O ≤ 4%). These data are typical of intraplate alkaline ocean island and guyot basalts^14^. The total tectonostratigraphic thickness in the Gorny and Malinovka Rivers interstream area is more than 3200 m and on the southern continuation of the structure west of the Meridionalny Fault it is more than 2000 m. The youngest (Tithonian) radiolaria are found in cherts of the complex’s lower horizons near the Koksharovka-Malinovka antiform axis^14,27^; that is, the overlying terrigenous rocks are the same age or younger, possibly Berriasian. The Tudovaka complex middle structural levels have chert of Triassic to Middle Jurassic age with overlying terrigenous rocks from the Middle–Late Jurassic age^41^. Thus, within the Tudovaka complex, the cherts show a downward younging trend from the upper to lower structural levels. It can be assumed that during complex’s formation, biogenic sedimentary rocks of the oceanic plate and overlying deep water trench distal turbidites were mainly accreted. Fragments of volcanic edifices in *sedimentary* slices appear only in the upper structural levels. It can also be noted that subduction occurred without any hindrances, since synsedimentary mélange is not characteristic of this complex.

#### The Ust’-Zhuravlevka complex

 (“The Samarka Formation”) is mapped by the mass appearance of hydrotectonites and synsedimentary terrigenous mélange (olistostrome). The mélange matrix consists of siltstone, deformed to varying degrees, sandstone layers alternating with hundred meter thick packages of arkose sandstone and, locally, lenses of gravelite and conglomerate. Evidence from radiolarian fossils indicates that the terrigenous matrix age ranges from Middle to Late Jurassic^39,42,43^. Inclusions are comprised of lenticular, tabular and isometric fragments of Triassic-Jurassic and rare Carboniferous-Permian rocks, slices and blocks of cherts, basalts, less common lumps of Carboniferous-Permian limestones, granitoids and banded gabbroids of uncertain age^14,27^. The proportions of different rocks vary from place to place. The true stratigraphic thickness of the Triassic-Lower Jurassic chert slices does not exceed 70 m^36^. Due to intense folding of the section during accretion, chert slices of this age can be many hundreds of meters thick. As distinct horizon markers, these cherts can be traced along strike for distances up to 5–10 km (Fig. 4). The total thickness of the complex is up to 4000 m.

The formation of hydrotectonites and olistostrome complexes is related to the deformation of rocks, including those just deposited, in the deep-water trench as they are sucked into the subduction zone. Thus, most of them can be called subduction-related mélange^14,15,43^. Intense mélanging of rocks of the Ust’-Zhuravlevka complex is due to seamounts (guyots) and their carbonate “caps” (represented as exotic blocks of basalt and limestone) significantly hindering subduction during the complex’s formation.

### The Udeka complex

It is made of greenish-gray, fine-grained, flaggy sandstone alternating with siltstone and is up to 1000 m thick. All contacts with the underlying Ust’-Zhuravlevka complex or overlying ophiolites of the Sebuchar complex are tectonic. This sandstone sequence is an excellent reference horizon that separates the abovementioned complexes at significant distances of more than 200 km (Fig. 3).

The clastic sandstone is composed of quartz and feldspar. The basal sandstone cement consists of chlorite and clayey-chlorite material. Heavy minerals in some sections of the Udeka sandstones have elevated (10–87%) amounts of pyroxene and olivine^44^. These sandstones were likely derived from the previously accreted, ophiolite allochthons overlying the Sebuchar complex. This is also supported by the sandstone chemical composition being near quartz-free diorites^44^. The age of these rocks is Middle to Late Jurassic based on radiolarian fossils^45^.

Thus, Udeka complex was formed during a relatively calm episode of Middle to Late Jurassic terrigenous sedimentation near blocks of previously accreted Sebuchar complex ophiolites.

### The Sebuchar complex

**Figure 5:**
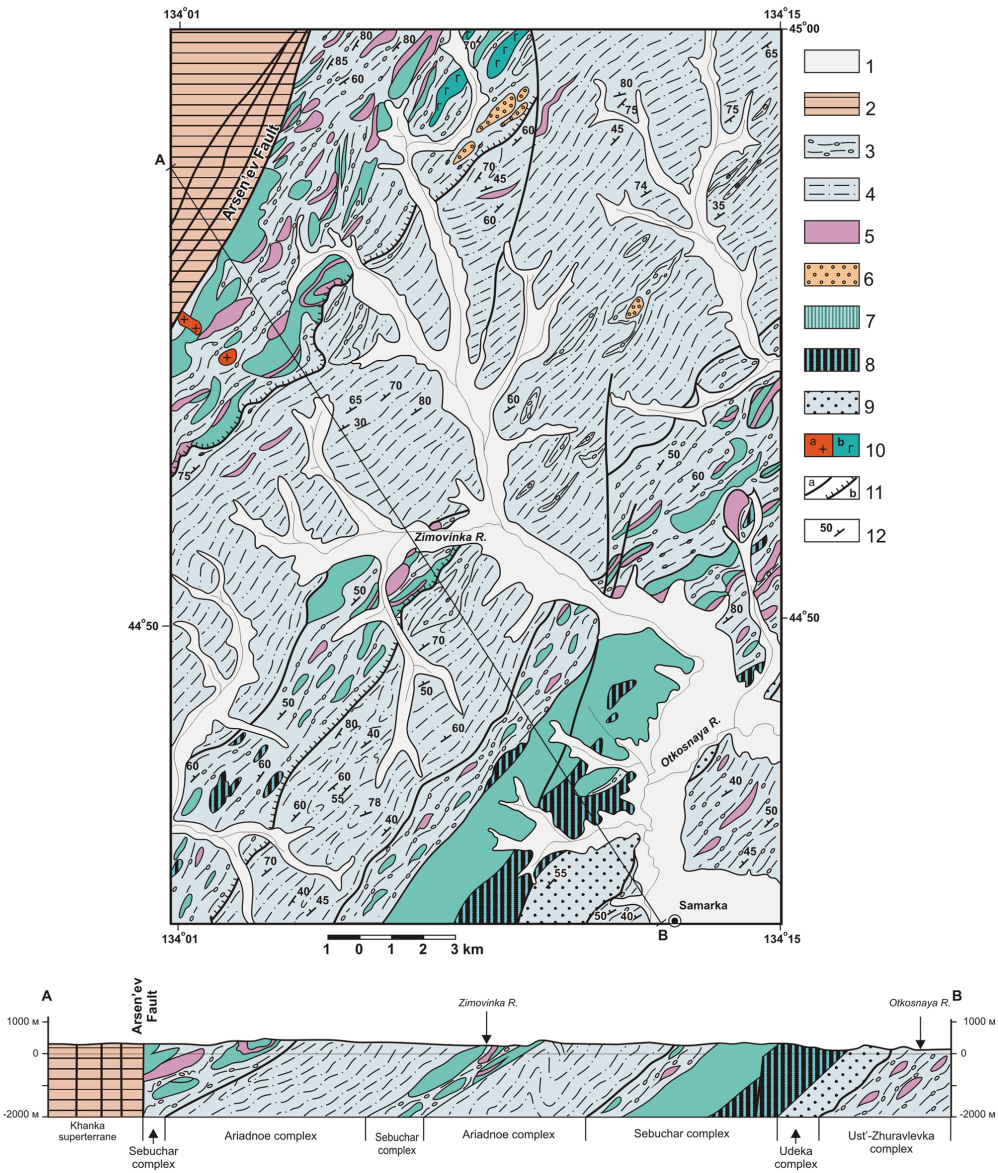
Upper structural levels of the Samarka terrane on the right bank of the near-mouth part of the Zhuravlevka River. Modified after [^14^, fig. 10]. Location see in the Fig. 3. 1 – Quaternary alluvium; 2 – Upper Permian and Mesozoic cover complex of the Khanka terrane; 3 – mixtite with siltstone matrix, included the sandstone interbeds and blocks and slices of sandstone and chert, more rarely –basalt and limestone; 4 – sandstone, siltstone and siliceous tuffite; 5 – chert; 6 – Permian tuffaceous terrigenous rock; 7–8 – Kalinovka ophiolite: 7 – basalt; 8 – gabbro and pyroxenite; 9 – sandstone–siltstone alternation; 10 – Early Cretaceous granite (**a**), gabbro (**b**); 11 – faults: a – strike-slip fault, b – thrust; 12 – bedding.

Figure 5 includes the Kalinovka ophiolite slices overlain by siliceous-volcanic-terrigenous formations that were previously identified as the Sebuchar Formation^46,47^.

#### Kalinovka ophiolites

**Figure 6:**
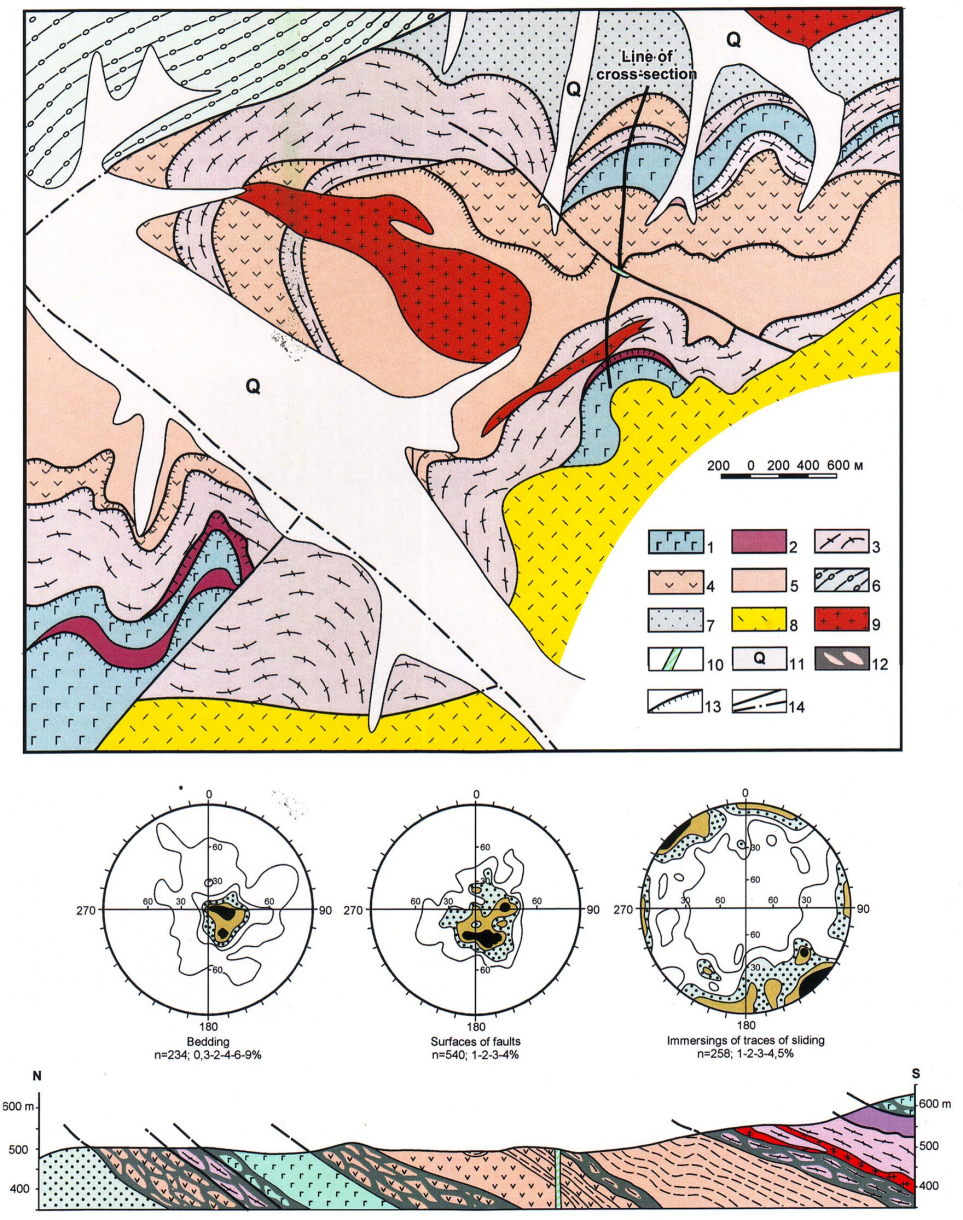
The bottom part of Sebuchar complex in the near-axial portion of the Chuguyevka synform. Modified after [^27^, fig. 14]. Location see in the Fig. 3. 1–5 – Sebuchar complex: 1 – gabbroid, 2 – serpentinized pyroxenite, 3 – mica schist and amphibolite, 4 – basalt, 5 – siltstone; 6 – Ust’-Zhuravlevka complex: siltstone with inclusions and slices of the sandstone and chert; 7 – Udeka complex: sandstone with the interbeds of the siltstone; 8–10 – Late Cretaceous volcanics (8), granite (9) and diabase (10); 11 – Quaternary alluvium; 12 – mylonite zone; 13 – thrust; 14 – normal and strike-slip faults.

It form an allochthonous sheet that can be traced for approximately 200 km from the right bank of the Otkosny River in the north to the settlement of Breevka in the south. The allochthonous ophiolites are localized in the wings and the core of a series of synforms and antiforms. They are composed of cataclazed, banded gabbroids and less common ultramafic rocks, basaltoids and cherts^14,27^ (Fig. 3). All ophiolites tectonically overly sandstones and siltstones of the Udeka complex. In all studied cases, the allochthon sole and tectonic schistosity of ophiolites are oriented subparallel to bedding of the underlying and overlying stratified formations. At the core of the Chuguevka synform, the allochthon sole retains its original low-angle bedding (Fig. 6). The thrust zone of this area is comprised of three tectonic sheets. The lower sheet (approximately 700 m thick) is composed of green schist milonite, gabbroids, crystalline schists, amphibolites and unmetamorphosed siltstone of unknown age; the middle sheet (approximately 150 m thick) consists of crystalline schists and amphibolites. The upper sheet represents the major volume of allochthon and is made up of gabbroids with lesser amounts of serpentinites. Almost all gabbroids are cataclazed and amphibolized to some extent.

Intraplate ophiolites and basalts are geochemically characterized by enriched iron and titanium (up to 4.2% TiO_2_)^9^. They are assumed to have been emplaced at the base of oceanic plateaus by mantle plumes^48^. Available data estimate the ophiolites age as Devonian to Early Permian^14,27^.

#### Siliceous-volcanic-terrigenous formations

It overlap and laterally replace sheets of the Kalinovka ophiolites. Their composition is largely similar to the rocks of the Ust-Zhuravlevka complex described above. They are made up of a somewhat tectonized siltstone matrix with blocks and sheets of cherts, basalts, rare gabbroids and limestones containing Carboniferous to Lower Permian foraminifera. Middle-Late Jurassic radiolarians were found in the siltstone matrix at lower structural levels^45^. The upper level matrix is an alternation of siltstone and siliceous tuffite containing Lower and Middle Jurassic radiolarians^15^. The allochthonous blocks and sheets consist of basalts, siltstone with Late Permian radiolarians, Carboniferous-Permian limestone and rare cherts of Middle and Late Triassic age. Jurassic cherts were not found at this structural level. These data make us confident in the older age of the matrix and allochthonous bodies relative to the underlying Udeka, Ust-Zhuravlevka and Tudovaka rock complexes. The total thickness of the tectonostratigraphic sequence of this complex is at least 4000 m.

Thus, it can be assumed that the fragments of the late Paleozoic seamounts, overlapping limestone caps and volcanic-terrigenous blocks from seamount slopes were accreted first during the formation of the Sebuchar complex. To a lesser extent, the complex includes Triassic allochthonous bodies of cherts and basalts. The final episode of the Sebuchar complex formation was an accretion of a Paleozoic oceanic plateau (Kalinovka ophiolites).

### The Okrainka-Sergeevka allochthonous complex

It is exposed in the study area’s southeastern territory adjacent to the Central Sikhote-Alin fault. It consists of continental basement rocks (Precambrian to early Paleozoic gabbroids with fewer granitoids and metamorphic rocks) overlain by a sedimentary-volcanic cover of Devonian, Permian, Triassic and Jurassic ages. These rocks are part of large allochthonous sheets in the Samarka accretionary prism and experienced all the deformations that took place in the prism during the Middle to Late Jurassic^14,27^. In the Zhuravlevka River basin, the thickness of basal gabbroid in thrust sheets range from 200 to 500 m, and the thickness of terrigenous, mainly shallow marine late Permian, Triassic, and Jurassic cover, reaches 1500 m. In the Partisansky River basin to the south, the complex thickness increases to several thousand meters due to thickening of both the basement gabbroids, granitoids and metamorphic rocks, and the late Permian sedimentary cover.

**Figure 7:**
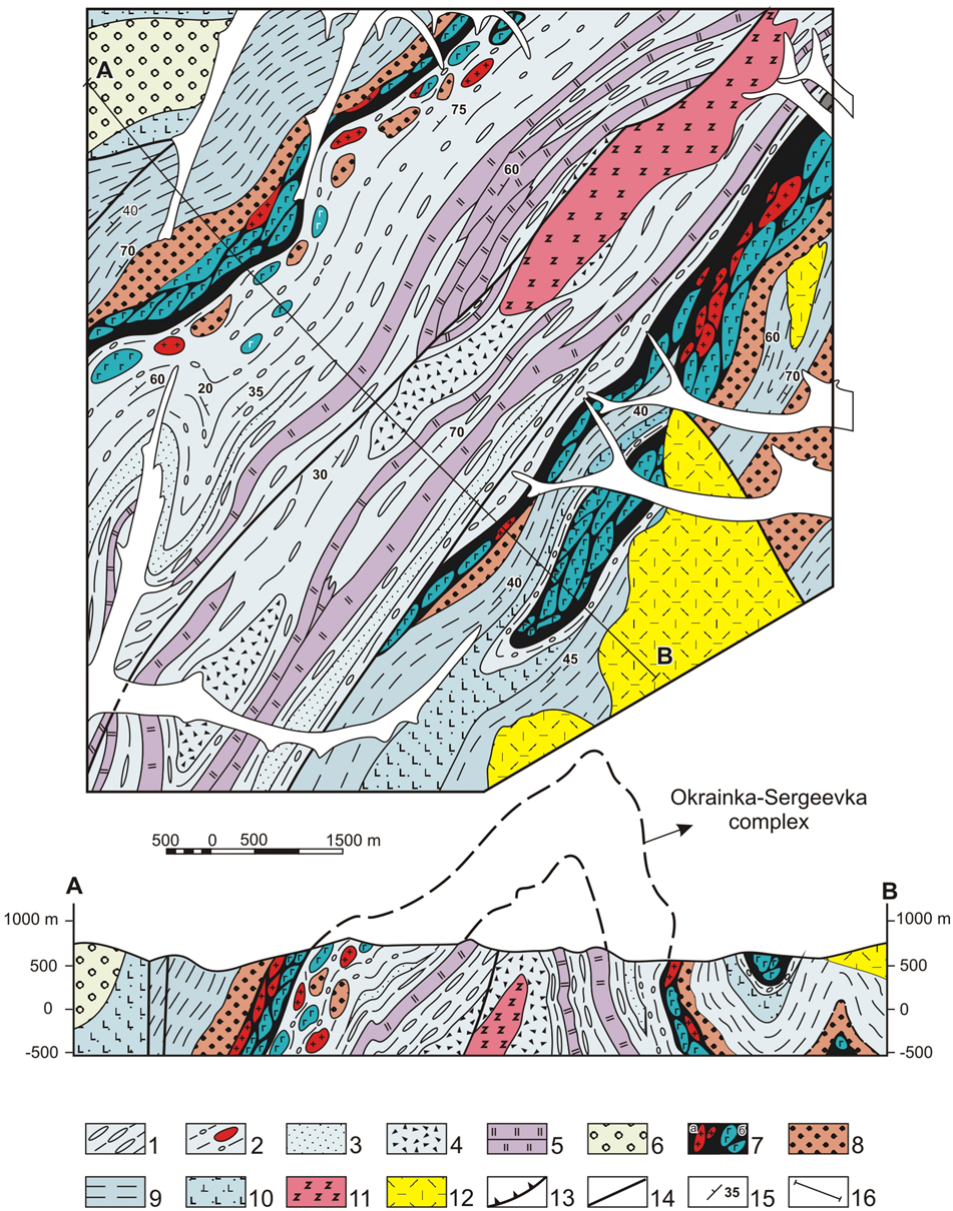
Fragments of the Okrainka-Sergeevka complex in the Vasil’ev antiform (explanations in the text). Modified after [^27^, fig. 11]. Location see in the Fig. 3. 1–5 – para-autochtone (Ust’-Zhuravlevka complex): 1 – hydrotectonite with siltstone matrix, interbeds and slices of the sandstone and chert, 2 – siltstone with the fragments, blocks and slices inclusions of the sandstone and chert, more rarely – gabbroid, granitoid, metamorphic rocks and Upper Permian micas sandstone, 3 – sandstone, 4 – sedimentary breccia, 5 – chert; 6 – neo-autochtone: Valanginian sandstone and gravelite; 7–10 – allochthone (Okrainka-Sergeevka complex): 7 – tectonized granitoid (a), and gabbroid (б), 8 – Upper Permian micas sandstone, 9 – Triassic-Middle Jurassic siltstone with the sandstone interbeds, 10 – Upper Jurassic-Berriasian alcaline basalt; 11 – Early Cretaceous nepheline syenite; 12 – Late Cretaceous volcanics; 13 – thrust; 14 – normal and strike-slip faults; 15 – bedding; 16 – line of the cross-section.

Direct contacts of Okrainka-Sergeevka complex allochthonous sheets with underlying terrigenous rocks of the Udeka or Ust-Zhuravlevka complex autochthon were observed in the Pavlovka River basin. An indicative example can be found in the Sinyaya River basin (the right tributary of the Pavlovka River), where the Vasilievsky antiform and conjugate Shumninsky and Pogranichny synforms are mapped^14,27^ (Fig. 7). The antiform is an inclined fold with a steeply dipping (60°–80°) southeast limb and gentler dipping (20°–50°) northwest limb. Its core exposes a more than 1600 m thick para-autochthon composed of siltstones with boudinaged blocks and slices of sandstones and cherts, sedimentary breccias and clumpy mixtites.

The allochthon is exposed on both limbs of the antiform and consists of cataclazed gabbroids and granitoids of the basement of the described complex (a thrust zone sensu stricto). The basement rocks are overlain unconformably by Upper Permian micaceous sandstones and siltstones, Triassic to Middle Jurassic siltstones with interlayers of sandstones and alkali basaltic tuffs of the Middle-Upper Jurassic Poga Formation^14,27,28^. The total thickness of these formations is approximately 1100 m. Within the study area, the Upper Permian and Upper Triassic successions are well documented by index fossils.

The sheets of cataclazed gabbroids and granitoids present on both limbs of the antiform have a similar structure and thicknesses ranging from 80 to 500 m. The sheet segments directly adjoining the underlying olistostrome horizon are 40 to 150 m thick milonite blocks with a round or oval shape, densely covered with furrows and gliding planes. Among them are small fragments and clumps of cataclazed crystalline schists, quartzites and Upper Permian micaceous crinoid-containing sandstones set in siltstone binder material. Large blocks gradually become sparser, giving way to a fine-grained matrix. Thus, the fault zone transitions to the olistostrome^14,27,28^.

In the core of the Shumninsky synform, located southeast of the Vasilievsky antiform, the allochthonous block is overlain by an olistostrome horizon made of siltstones with fragments and blocks of sandstones, cherts and basaltic tuffs. This suggests that the allochthonous sheet was “sealed” by newly formed sediments. The upper olistostromic sequence is thin, 50–100 m, and is overlain by an allochthonous sheet composed of banded gabbroids.

### The Ariadnoe complex

It consists almost exclusively of alternating sandstone and siltstone terrigenous rock reaching 1,700 m thickness and united into the Formation under the same name. Some sandstones contain siliceous tuffite intercalations with remains of Jurassic radiolaria^45^. The complex roof includes a 750 m thick sequence of mainly siltstones. These siltstones are interlayered with rare siliceous tuffites and mixtites. The mixtites represent poorly sorted siltstones contaminated with different-grained sandy material and inclusions of sandstones, basalts, cherts and siliceous tuffites. This sequence also contains Jurassic radiolarians and a matrix which includes blocks and lumps of cherts, basalts volcano-terrigenous rocks and limestone-siliceous rocks of Carbon-Permian age^45^. In addition, individual exotic blocks are tuffoconglomerates that contain the remains of Late Permian bryozoans and crinoids^4^. Lumps and slices of primarily Triassic cherts, which are common in the above-described Sebuchar, Ust-Zhuravlevka and Tudovaka complexes, have not been reliably confirmed at this structural level.

## Discussion

**Figure 8:**
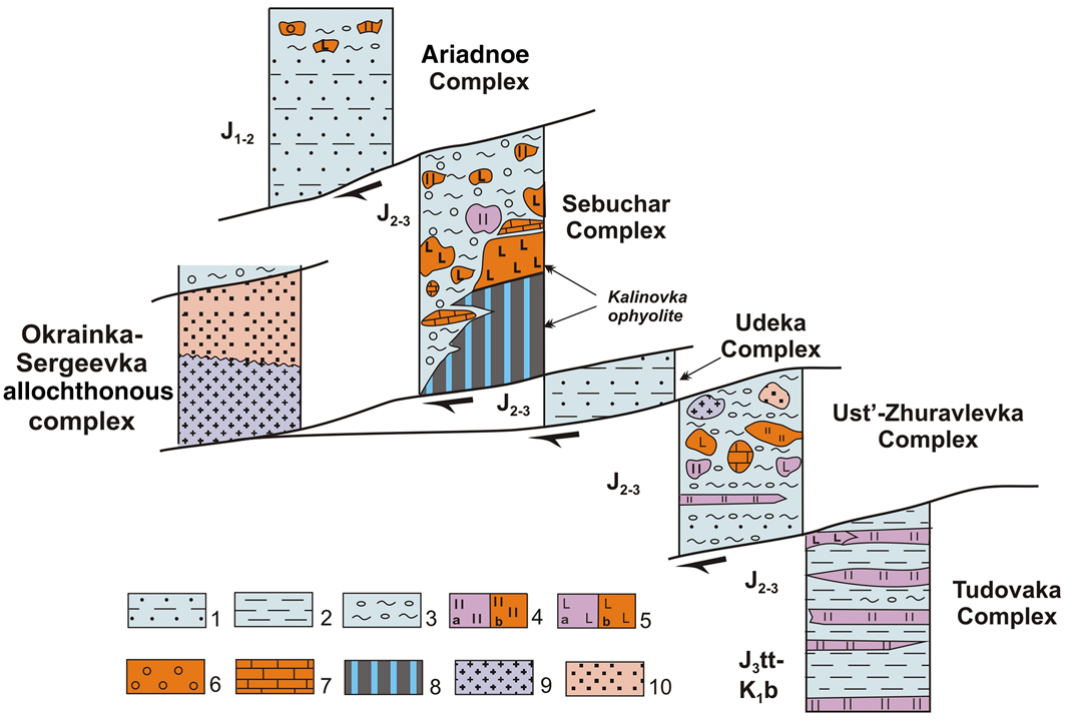
Relationships of tectonostratigraphic complexes in the southern part of the Samarka Terrane, a fragment of the Jurassic accretionary prism. 1–3 – matrix of accretionary prism: 1 – sandstones, siltstone, 2 – siltstone, 3 – mixtite. The radiolarian age of the matrix is shown by indexes  to the left of the columns. 4–8 – composition of blocks and allochthonous sheets: 4 – Triassic-Jurassic chert (**a**), Carboniferous-Permian chert (**b**), 5 – Triassic-Jurassic basalt (**a**), Carboniferous-Permian basalt (**b**), 6 – Late Permian tuff conglomerates, 7 – Carboniferous-Permian limestones, 8 – Middle Paleozoic gabbroids; 9–10 – Okrainka-Sergeevka complex – a fragment of an overhanging continental plate, including Pre-Permian basement (9) and a cover of the Late Permian, Triassic and Jurassic (10).

The tectonostratigraphic complexes’ age relationships discussed above are summarized in Fig. 8. They demonstrate a clear downward younging age trend in both the matrix and allochthonous bodies from the upper to lower structural levels. Thus, the model of Samarka accretionary prism formation by successive underthrusting of increasingly younger fragments of the oceanic plate during subduction may be considered proven.

The proposed scheme for subdividing the Samarka terrane, a Jurassic accretionary prism fragment, into tectonstratigraphic complexes provides a basis to study and map these formations at a medium- to large-scale. Here, each of the complexes corresponds to a certain stage of prism formation. Thus, the complexes composed of subduction mélange and olistostromes (e.g., the Ust-Zhuravlevka and Sebuchar complexes) can be correlated to episodes when the underthrusting of seamounts hampered subduction, as evidenced by seamount fragments contained in the complexes. There are also episodes of relatively quiet subduction, resulting in complexes composed mainly of normally bedded terrigenous and biogenic formations (e.g., the Tudovaka, Udeka and partially Ariadnoe complexes).

It is important to note that these complexes can be traced over considerable distances. In particular, the extensions of the Ust-Zhuravka, Udeka and Sebuchar complexes are mapped to the north in the Bikin River basin. This basin contains allochthonous sheets of hyperbasites, gabbros and basalts of the Takholo complex that are analogous to the Kalinovka ophiolites^49^. Other analogs of the Kalinovka ophiolites are the Dahengen ophiolites (Nadanhada terrane in northeastern China) and the Yakuno ophiolites of the Ultra-Tamba terrane, which is a fragment of the Jurassic accretionary prism of southwestern Japan^15^.

### Structural relationships between Sebuchar and Okrainka-Sergeevka complexes

The sheets composed of the Okrainka-Sergeevka and Sebuchar complex formations, as already mentioned, almost always overlie sandstones and siltstones of the Udeka complex. We conclude that the Okrainka-Sergeevka and Sebuchar complexes are at almost the same structural level in the accretionary prism and replace each other laterally. Where seen together, the Kalinovka ophiolites (for example, outcrops of troctolite and dunite near the village of Vladimiro-Aleksandrovsky) compose separate sheets emplaced among gabbroids and granitoids of the Okrainka-Sergeevka complex.

**Figure 9:**
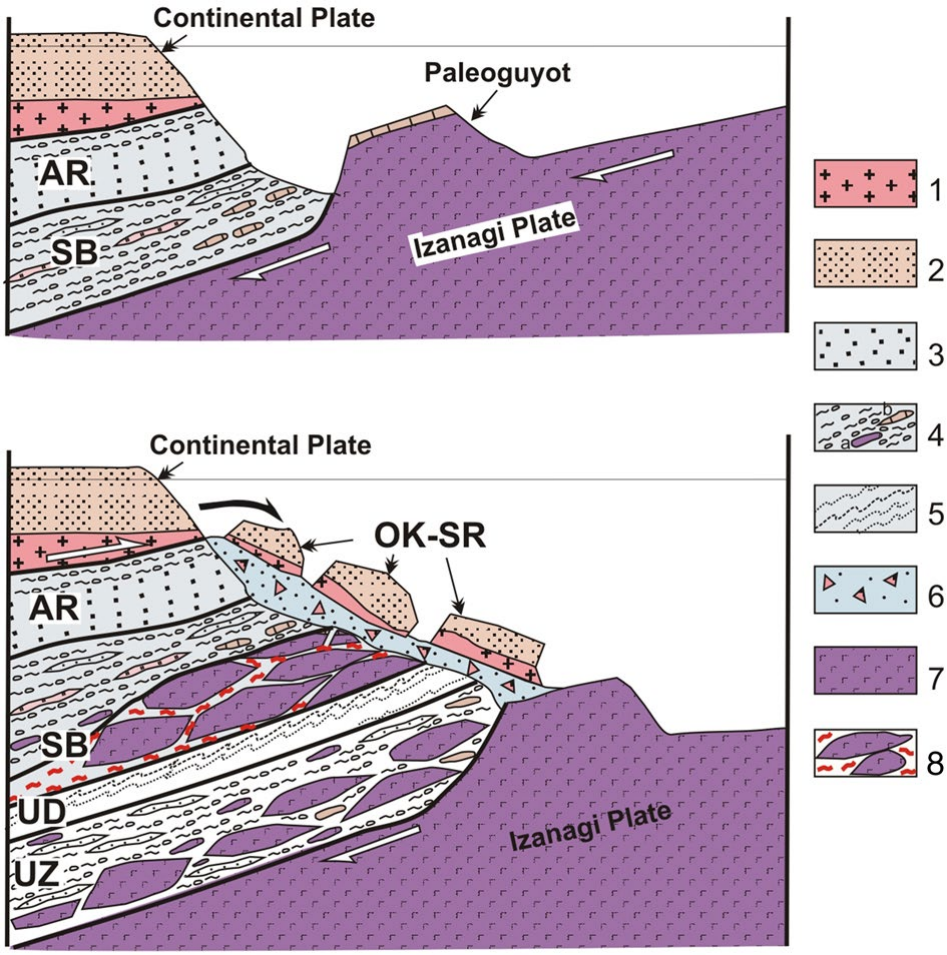
The model of “tectonic moraine” explaining the location of allochthnous sheets of continental and oceanic rocks at closely spaced structural levels (explanations in the text). 1–2 – continental plate, including: 1 – basement (Pre-Permian gabbroid, granitoid and metamorphic rocks, 2 – cover of the Late Permian, Triassic and Jurassic age; 3–5 – Jurassic accretionary prism, including: 3 – sandstone with interlayers of siltstone, 4 – hidrotectonite and olistostrome with a silty matrix containing inclusions, lumps and sheets of cherts and basalts (**a**), limestones (**b**); 5 – sandstone and siltstone alternation; 6 – olistostrome; 7 – oceanic plate; 8 – zones of crushing of the paleoguyot. Tectonostratigraphic complexes: AR – Ariadnoe, SB – Sebuchar, OK-SR – Okrainka-Sergeevka, UD – Udeka, UZ – Ust’-Zhuravlevka.

The Okrainka-Sergeevka complex appears to be a fragment of Precambrian-Early Paleozoic continent hanging over a subduction zone^14^. Thrusting and movement of allochthonous sheets toward the trench apparently occurred, with partial jamming of the subduction zone during accretion of the oceanic plateau. The Kalinovka ophiolites are part of this zone. Under the unceasing pressure of the oceanic plate, the “weakest link” could be a décollement zone at the base of the continental plate sedimentary cover. Fragments of this plate that were pushed toward the surface gravitationally slid down into the deep-sea trench (Fig. 9). In this scenario, it is understandable that the Sebuchar and Okrainka-Sergeevka complexes are at a similar structural level.

### On the possible genetic relationship of hydrotectonites and olistostromes with gravitational sliding of allochthonous sheets

As seen above, formation of the Jurassic Samarka accretionary prism terrane was not always accompanied by cosedimentary dislocations, evidenced by presence of hydrotectonites and olistostromes.

The current sequence of structural units in the Sinyaya River basin (see the map and geological sections in Fig. 7) consists of two overlapping allochthonous sheets separated by the olistostrome horizon. The lower sheet, which has the largest distribution, is composed of Late Proterozoic basement mylonites after gabbroids and granitoids, and Late Permian, Triassic and Jurassic platform cover. This sheet is underlain by the olistostrome horizon, which contains blocks of gabbroids, granitoids and late Permian sandstones derived from the sheet destruction. Below, the olistostromes are replaced by hydrotectonites. At lower levels of the section, a chaotic structure gradually lessens, inclusions of exotic rocks disappear and less deformed layers of cherts and sandstones are seen. These relatively weakly tectonized formations are underlain by the next olistostrome horizon of sedimentary breccias with fragments of the same gabbroids, granitoids and crystalline schists. Thus, the described complexes are somewhat rhythmic, which can be related to the movement of allochthonous sheets. First, there is no doubt that gravitational load of the moving sheet affected the partly diagenesized autochthonous sediments. This influence depended on the sheet thickness and its affect gradually weakened down the autochthon section. Second, the underlying sediments were involved in the movement, and further crushing and rolling of boudins and rock inclusions occurred. Thus, the observed rhythmicity in the complexes’ structure can be explained by the pulsating movement of allochthonous blocks. During calm periods, relatively thick horizons of sediments had time to accumulate.

### Accretionary prism of the Samarka terrane – a fragment of nonvolcanic subduction zone

An important feature of the Samarka accretionary prism and its analogs located in Northeast China and Japan is that volcanics are present only in allochthonous material and are represented by N-MORB and OIB basalts, with a predominance of the latter^9,14,21,27^. The autochthon matrix is characterized by a lack of tuff and tuffite horizons, products of synchronous suprasubduction volcanism. The terrigenous Samarka terrane matrix is free of heavy minerals characteristic of volcanic arcs^50^. Thus, the absence of both suprasubduction volcanism and an early Jurassic-Berriasian (180–142 Ma) volcanic belt contemporaneous with the Samarka accretionary prism and its analogs suggest the terranes formed at a nonvolcanic subduction zone^14,51,52^.

Some sections of the Andean margin provide modern examples of nonvolcanic subduction zones. Suprasubduction volcanic belts are absent in cases of flat-slab subduction due to the absence of an asthenospheric wedge between the slab and the continental plate. Flat-slab subduction arises when a thickened, low-density, and therefore floating lithosphere (aseismic ridges, seamounts, etc.), is subducted^34,53–57^. In this situation, subduction zones slopes become gentler, sometimes close to horizontal. During flat-slab subduction, small magmatic complexes are formed in the continent’s interior above the area where the slab bends and ruptures. These complexes are geochemically distinct from typical suprasubduction complexes^55,58^. We can confidently assume a thickened oceanic plate once subducted under the Eurasian continent during the Jurassic because the Samarka terrane contains fragments of a large, Late Paleozoic, plume-related oceanic plateau. This is represented by the Kalinovka ophiolites which have variable-depth gabbro-hyperbasite associations and high-Ti and high-Fe basalts. The Samarka terrane also contains blocks and sheets of OIB-type basalts in the Tudovaka, Ust-Zhuravlevka and Sebuchar complexes.

## Conclusions

1. The proposed scheme to subdivide the Samarka terrane, a Jurassic accretionary prism fragment, into tectonstratigraphic complexes provides a basis for studying and mapping these formations at a medium- to large-scale. Here, each complex corresponds to a certain stage in the prism formation. Thus, the complexes composed of subduction mélange and olistostromes (e.g., the Ust-Zhuravlevka and Sebuchar complexes) can be correlated to episodes when the underthrusting of seamounts hampered subduction, as evidenced by seamount fragments contained in the complexes. There are also episodes of relatively quiet subduction, resulting in complexes composed mainly of normally bedded terrigenous and biogenic formations (e.g. the Tudovaka, Udeka and partially Ariadnoe complexes). The Okrainka-Sergeevka allochthonous complex, a fragment of continental plate hanging over a subduction zone that was included in the accretionary prism during gravitational sliding on the internal slope of the paleotrench, is particularly considered.
2. Volcanic rocks in the accretionary prism are all allochthonous fragments of the accreted oceanic crust. The absence of the Jurassic-Berriasian volcanic belt related to this prism, as well as synchronous autochthonous volcanism, indicates that the accretionary prism of the Samarka terrane formed under conditions of flat-slab subduction, similar to modern examples along the Andean margin.
